# Microbial keratitis in Southern Malawi: a microbiological pilot study

**DOI:** 10.1136/bmjophth-2024-001682

**Published:** 2024-04-22

**Authors:** Tobi F Somerville, Shaffi Mdala, Thokozani Zungu, Moira Gandiwa, Rose Herbert, Dean Everett, Caroline E Corless, Nicholas A V Beare, Timothy Neal, Malcolm J Horsburgh, Alistair Darby, Stephen B Kaye, Petros C Kayange

**Affiliations:** 1 Department of Eye and Vision Sciences, University of Liverpool, Liverpool, UK; 2 Queen Elizabeth Central Hospital, Blantyre, Southern Region, Malawi; 3 Ophthalmology Unit, Kamuzu University of Health Sciences, Blantyre, Southern Region, Malawi; 4 Kamuzu Central Hospital, Lilongwe, Central Region, Malawi; 5 Malawi-Liverpool-Wellcome Trust Clinical Research Programme, Blantyre, Malawi; 6 College of Medicine and Health Sciences, Infection Research Unit, Khalifa University, Abu Dhabi, UAE; 7 Medical Microbiology, Liverpool University Hospitals NHS Foundation Trust, Liverpool, UK; 8 University of Liverpool, Liverpool, UK; 9 Department of Infection and Immunity, Liverpool University Hospitals NHS Foundation Trust, Liverpool, UK; 10 Department of Infection Biology and Microbiomes, University of Liverpool, Liverpool, UK

**Keywords:** Cornea, Infection, Treatment Medical

## Abstract

**Objective:**

Microbial keratitis (MK) is a significant cause of blindness in sub-Saharan Africa. We investigated the feasibility of using a novel corneal impression membrane (CIM) for obtaining and processing samples by culture, PCR and whole-genome sequencing (WGS) in patients presenting with suspected MK in Malawi.

**Methods and analysis:**

Samples were collected from patients presenting with suspected MK using a 12 mm diameter polytetrafluoroethylene CIM disc. Samples were processed using culture and PCR for *Acanthamoeba*, herpes simplex virus type 1 (HSV-1) and the bacterial 16S rRNA gene. Minimum inhibitory concentrations of isolates to eight antimicrobials were measured using susceptibility strips. WGS was used to characterise *Staphylococcus aureus* isolates.

**Results:**

71 eyes of 71 patients were included. The overall CIM isolation rate was 81.7% (58 positive samples from 71 participants). 69 (81.2%) of isolates were Gram-positive cocci. Coagulase-negative *Staphylococcus* 31.8% and *Streptococcus* species 14.1% were the most isolated bacteria. Seven (9.9%) participants were positive for HSV-1. Fungi and *Acanthamoeba* were not detected. Moxifloxacin and chloramphenicol offered the best coverage for both Gram-positive and Gram-negative isolates when susceptibility was determined using known antimicrobial first quartile concentrations and European Committee on Antimicrobial Susceptibility Testing breakpoints, respectively. WGS identified known virulence genes associated with *S. aureus* keratitis.

**Conclusions:**

In a resource-poor setting, a CIM can be used to safely sample the cornea in patients presenting with suspected MK, enabling identification of causative microorganisms by culture and PCR. Although the microbiological spectrum found was limited to the dry season, these preliminary results could be used to guide empirical treatment.

**WHAT IS ALREADY KNOWN ON THIS TOPIC**

- Corneal ulceration due to microbial keratitis (MK) is a significant cause of blindness in sub-Saharan Africa; however, a lack of specialised equipment and training in obtaining corneal samples means causative organisms are rarely identified and treatment is poorly targeted.

**WHAT THIS STUDY ADDS**

- This study used a novel, simple-to-perform corneal sampling method to characterise the microbiological profile of MK in Malawi for the first time, with the causative microorganisms identified largely as Gram-positive bacteria. Preliminary susceptibility data showed moxifloxacin and chloramphenicol to offer the best antimicrobial coverage and whole-genome sequencing of *Staphylococcus aureus* isolates identified known virulence factors in MK to be present in this population.

**HOW THIS STUDY MIGHT AFFECT RESEARCH, PRACTICE OR POLICY**

- This study demonstrates the potential utility of the corneal impression membrane as a sampling modality in clinical practice and future research studies in Malawi and similar resource-poor settings. Although the findings are limited to the dry season in Malawi, the microbiological profile, analysis of susceptibility to commonly used ophthalmic antimicrobials and identification of virulence factors will help inform MK treatment decisions in this population.

## Introduction

Corneal ulceration due to microbial keratitis (MK) is recognised as one of the leading causes of blindness worldwide.^1^ The incidence, prevalence and burden of disability of MK in low- and middle-income countries (LMICs) are under-reported.^2^ The population incidence of MK in LMIC has been estimated by two studies from the tropics to range annually between 113 and 362 per 100 000,^3 4^ which is at least 10 times higher than reported in North America and Europe.^5 6^

The microbiology of MK in Malawi is unknown and clinical microbiology facilities are limited, meaning only presumptive antimicrobial treatments can be given, usually a broad-spectrum antibacterial if available. Studies from several other African countries have shown a diverse aetiology associated with MK from viral causes such as herpes simplex virus type 1 (HSV-1) to a variety of fungal and bacterial species.^7–12^ There is, therefore, a need for data to enable the development of strategies to reduce the burden of MK and improve outcomes in sub-Saharan Africa.

One of the main barriers to identifying the causative microorganisms has been the difficulty in collecting samples from the affected area of the cornea. The standard method requires a slit lamp biomicroscope and expertise in its use. It also requires the collection of several samples from the corneal ulcer using sharp instruments such as a scalpel blade or needle, with care taken not to inadvertently deepen the ulcer or cause additional trauma. Samples are then plated on fresh microbiological agar plates. Isolation rates using this method remain relatively poor and the specialist equipment (biomicroscope, agar plates) and personnel who can perform corneal scrapes are often not available.

We have developed a method to sample a corneal ulcer which uses a corneal impression membrane (CIM) made from polytetrafluoroethylene (PTFE) that is placed directly on the corneal ulcer.^13 14^ This method, impression cytology, has been shown to reliably remove epithelial surface cells and infective organisms, increasing the detection of bacterial,^13 14^ viral,^15^ fungal^16^ and *Acanthamoeba* 17 microorganisms in cases of MK. This technique is simple to perform, less traumatic than the conventional corneal scraping method, increases the isolation and diagnostic rate^13–15^ and has been shown to promote prolonged stability and recovery of the microorganisms from samples.^18^ Importantly, it does not rely on using a slit lamp biomicroscopy by an ophthalmologist or suitably trained healthcare professional as the CIM is simply placed on the affected cornea for 2 s.

Samples can be transported in brain heart infusion (BHI) broth to the laboratory, where they are then plated and cultured. This does not require the direct plating of samples onto microbiological culture plates but has an equivalent isolation rate with no loss of sensitivity or specificity.^19^

The presence of bacterial virulence factors is an important determinant of clinical outcome, but their detection requires molecular techniques. For example, *Staphylococcus aureus* is one of the commonly isolated MK pathogens worldwide and disease outcomes are affected by the presence of virulence factors.^20^ *S. aureus* virulence and antimicrobial resistance factors are known to differ geographically and between infections and asymptomatic carriers and information available regarding these factors in Malawi is non-existent.^21^ We, therefore, used whole-genome sequencing (WGS) to determine the presence of key virulence factors in the Malawian *S. aureus* keratitis isolates that have previously been associated with ocular *S. aureus* infection.^22 23^

The aim of this study was to investigate the feasibility of using a CIM for obtaining and processing samples by conventional diagnostic culture, PCR and WGS in patients presenting with suspected MK in Malawi.

## Materials (subjects) and methods

### Participant recruitment

We recruited consecutive patients presenting with clinically suspected bacterial, fungal, viral or *Acanthamoeba* MK to Queen Elizabeth Central Hospital (QECH), an urban tertiary eye hospital, and Thyolo District Hospital, a rural secondary-level hospital in Malawi between April and July 2017 which is dry season. On presentation, demographic and clinical information were collected which included the type of treatment that the patient had received or was still using at presentation and risk factors such as trauma, contact lens wear, previous ocular surgery and previous MK. Best corrected visual acuity was measured using a Snellen chart and size, location and depth of the ulcer assessed using a slit lamp biomicroscope. Ulcer size (minor and major axis) and location (minimum distance from the limbus) were measured to the nearest 0.5 mm. The ulcer depth was graded on a nominal scale of 1–4 based on the proportion of corneal thickness lost on slit-lamp biomicroscopy (1: ≤25%, 2: 26%–50%, 3: 51%–75%; 4: 76%–100%).

### Sample collection

Three CIM samples and two conjunctival swabs were collected from the affected eye of each patient by ophthalmic clinical officers and ophthalmology registrars who were trained in the sampling methodology. A topical anaesthetic (preservative-free oxybuprocaine 0.4%) was installed into the patient’s lower conjunctival fornix. Using sterile gloves and with care to avoid contact with the eyelids or conjunctiva, a CIM (PTFE Millicell Cell Culture Insert, 12 mm, pore size of 0.4 µm; Merck Millipore, UK) was placed over the ulcer area for 2 s. One CIM was placed immediately into BHI broth and two further CIMs were placed into empty sterile bijou tubes. Two conjunctival swabs (Sigma Transwab and Sigma Virocult, MWE, UK) were obtained by sweeping the swab over the patient’s lower fornix four times. Samples from Thyolo District Hospital were transported 40 km at room temperature by car and delivered to the Malawi-Liverpool-Wellcome Trust (MLW) laboratory within 50–60 m. Samples collected at QECH were transported to the MLW laboratory by hand, typically arriving within 5 m. On arrival at the MLW laboratory, the two dry CIMs and Virocult swab were immediately stored at −76°C. We have previously demonstrated there to be no significant reduction in microorganism DNA recovery when CIMs were stored for 3 days at temperatures up to +35°C.^18^

### Conventional diagnostic culture

The BHI bottle and transwab were vortexed for 5–10 s and 10 µL inoculated onto blood, chocolate and Sabouraud’s dextrose agar plates. Blood and chocolate agar plates and the BHI broth were incubated at 35–37°C and Sabouraud’s dextrose agar plates were incubated at 30°C. Agar plates and a 24-hour subculture of the BHI broth were examined for evidence of bacterial growth after 24 hours and 48 hours of incubation. Sabouraud’s dextrose agar plates were examined daily for 14 days for any growth. All isolates were identified using matrix-assisted laser desorption ionization-time-of-flight mass spectrometry (Bruker, Bremen, Germany).

At a later date, bacterial minimum inhibitory concentrations (MICs) were determined for *S. aureus*, *Streptococcus* and Enterobacteriaceae isolates using susceptibility strips containing a concentration gradient of the following antimicrobials: ciprofloxacin, ofloxacin, moxifloxacin, cefuroxime, gentamicin, vancomycin, chloramphenicol and teicoplanin (BioMérieux SA, Marcy l'Etoile, France). Isolates were plated onto blood agar and incubated for 24 hours. Discrete colonies were suspended in 0.85% (w/v) saline to create an inoculum with a turbidity of 0.5 McFarland units. The inoculum was replated onto Muller-Hinton agar (*S. aureus*, Enterobacteriaceae), or Muller-Hinton agar with 5% (v/v) horse blood (*Streptococcus*), susceptibility strips applied, and plates incubated in room air or 5% carbon dioxide at 37°C. After 24 hours the e-test strips were read. The American Type Culture Collection (American Type Culture Collection, Manassas, Virginia, USA) strains 29213 (*S. aureus*), 27853 (*Pseudomonas aeruginosa*), 49619 (*Streptococcus pneumoniae*), 29212 (*Enterococcus faecalis*) and 25922 (*Escherichia coli*) were used for quality controls. Cefoxitin (30 µg) discs were used to confirm methicillin resistance in *S. aureus*. If the MIC of the antimicrobial is below the known first quartile concentration of the antimicrobial in the cornea, then it is likely that the microorganism is susceptible^24–28^ (online supplemental tables S1–S3) . Bacteria were also deemed resistant if the measured MIC exceeded the breakpoint defined by the European Committee on Antimicrobial Susceptibility Testing (EUCAST).^29^ Topical breakpoints are available for ciprofloxacin, ofloxacin and chloramphenicol; for other antibiotics, systemic breakpoints were used to determine susceptibility.

10.1136/bmjophth-2024-001682.supp1 Supplementary data

### PCR

The remaining samples (two CIMs and one Virocult swab) were transported to the UK at −76°C for DNA extraction and PCR analysis. For all participants, targeted HSV-1 and *Acanthamoeba* PCR was performed. For those participants that had a negative culture, real-time 16S PCR was performed. On extraction, 400 µL of 4-(2-hydroxyethyl)-1-piperazineethanesulfonic acid (HEPES)-based buffering agent (Hologic Aptima, Marlborough, Massachusetts, USA) was added to the CIMs and Virocult swab in their primary tubes and vortexed for 10 s. After brief centrifugation, the 400 µL of HEPES-based buffering agent was transferred to a secondary tube. DNA was selectively extracted from the decanted HEPES using the Roche MagNA Pure Compact automated extraction platform and the MagNA Pure Compact DNA isolation kit I with a final elution volume of 50 µL (Roche Magna Pure Compact, Roche). A multiplex PCR master mix comprising of LC480 Probes Master, primers and fluorescently labelled probes (Eurogentec) for the detection of HSV-1 (Bennett *et al* 30) and *Acanthamoeba* (Qvarnstrom *et al* 31 and Rivière *et al* 32) was prepared according to methods we have previously described in Somerville *et al*.^18^ The cycle threshold (Ct) value, which is the PCR cycle at which the emitted fluorescence reaches a defined threshold, was recorded for each reaction. Based on the work of Bennett *et al*,^30^ and Rivière *et al*,^32^ a Ct value of less than or equal to 38.7 and 39.0 was set as the cut-off for a positive result for the HSV-1 and *Acanthamoeba* DNA amplification, respectively. For the detection of the bacterial 16S rRNA gene, a PCR master mix was prepared, and PCR was performed according to the methods we have previously described in Somerville *et al*.^33^ Samples that were found to have a Ct value of less than or equal to 31 were amplified and sequenced according to these methods.^33^

Statistical analysis was performed using SPSS (V.22). The χ^2^ test was used to test associations between MK risk factors and those patients with positive isolates.

### WGS of *S. aureus* keratitis isolates

The *S. aureus* keratitis isolates obtained in this study were cultured for genomic extraction by inoculating a single colony in Lysogeny broth (LB) and incubating at 37°C with shaking at 200 rpm overnight. Chromosomal DNA was extracted using the Qiagen DNeasy Blood and Tissue kit according to the manufacturer’s instructions. During the cell lysis step, samples were incubated at 37°C for 30 m with 5 µL lysostaphin (stock 2.5 mg/mL DNA) and mutanolysin (1 kU/mL). The concentration and purity of genomic DNA were measured using a Qubit dsDNA high-sensitivity assay and DNA quality was visualised by agarose gel electrophoresis. Genomic DNA was barcoded using the Nanopore Native barcoding genomic DNA protocol (Version: NBE_9065_v109_revAB_14Aug2019) and sequenced on MinION (ONT, Oxford, UK) using the R9 flowcell (FLO-MIN106; ONT, Oxford, UK). Raw sequence reads were base-called and demultiplexed using ONTs minKNOW software Guppy V.4.5.4. Sequence summary and read length histograms were generated using Nanoplot^34^ and SeqStat V.1.0.1. De novo assembly was conducted using default parameters in Canu.^35^ To assess the virulome, BLASTx was used to query known virulence genes^22 23^ associated with ocular *S. aureus* isolates against the sequenced genomes using a 90% threshold for %identity and e-value score <0.01 (online supplemental table S4).

### Patient and public involvement

Patient and public involvement informed the development of the CIM sampling method used in this study. As this was a pilot study, patients or the public were not involved in its design or conduct.

## Results

### Participants and ulcer characteristics

71 eyes of 71 participants with clinical suspected MK were included. Samples were obtained from 50 eyes of 50 participants recruited from QECH and 21 eyes of 21 participants from Thyolo District Hospital. The baseline demographics and predisposing risk factors are given in table 1 and the clinical characteristics of the ulcers are given in table 2. No problems were identified when transporting the CIM and swab samples from Thyolo District Hospital or QECH to the MLW laboratory.

**Table 1:** Baseline demographics and predisposing risk factors of 71 participants presenting with presumed MK

Age (mean, SD)	39.5 years (SD 19.8)
Male participants (%)	36 (50.7%)
Participants living in a rural place of residence (%)	44 (62.0%)
Participants working in agriculture (%)	27 (38.0%)
Time between symptom onset and presentation (median, IQR)	14 days (IQR 7–38.5)
Participants referred from another health facility (%)	21 (31.0%)
HIV positive (%)	12 (17.0%)
Previous history of MK (%)	9 (12.7%)
Trichiasis (%)	9 (12.7%)
Contact lens wear (%)	0
Previous corneal surgery (%)	0
Ocular trauma (%)	11 (15.5%)
Topical antimicrobial used immediately prior to presentation (%)	29 (40.5%)
Traditional medicine use (%)	26 (36.6%)

**Table 2:** Clinical characteristics of the ulcers of 71 participants presenting with presumed MK

Central location of ulcer (%)	53 (74.6%)
Distance from limbus (mean, SD)	2.0 mm (SD 1.5)
Major diameter of ulcer (mean, SD)	4.0 mm (SD 2.6)
Minor diameter of ulcer (mean, SD)	2.9 mm (SD 2.6)
Mean ulcer depth grade (mean, SD)	1.0 (SD 0.9)
Corneal perforation on presentation (%)	9 (11.3%)

### Conventional diagnostic culture

The overall CIM isolation rate using conventional diagnostic culture was 83.0% (59 positive samples from 71 participants). This compared with 17 (23.9%) from conjunctival swabs. One further *Neisseria gonorrhoeae* was isolated from a conjunctival swab that was not isolated using a CIM.

The CIM microbial isolates are shown in table 3. Gram-positive bacteria were the predominantly isolated organisms. Coagulase-negative *Staphylococcus* (CNS) and other Gram-positive species were the most commonly isolated bacteria. No fungal growth was seen. Mixed growth was seen in 18 (25.4%) of the CIM samples (table 4). CNS were found in 11/16 (68.8%) of the mixed samples with one sample having two types of CNS species present.

**Table 3:** Isolates from CIMs of cases of presumed MK

	Isolates, n (%) (N=88)
Bacteria*	
Total Gram-positive	71 (80.7%)
*Staphylococcus aureus*	7 (8.0%)
*Streptococcus pneumoniae*	4 (4.5%)
Other *Streptococcus* spp†	11 (12.5%)
CNS‡	26 (29.5%)
Other Gram-positive bacteria§	23 (26.1%)
Total Gram-negative	9 (10.2)
*Morganella morganii*	3 (3.4%)
*Enterobacter cloacae*	1 (1.1%)
*Serratia marcescens*	1 (1.1%)
*Klebsiella variicola*	1 (1.1%)
*Citrobacter youngae*	1 (1.1%)
*Sphingomonas paucimobilis*	1 (1.1%)
*Aeromonas caviae*	1 (1.1%)
Fungi¶	0
Viral	
HSV-1**	8 (9.1%)
Protozoa	
Acanthamoeba**	0

*Bacterial isolates identified using culture and MALDI-TOF.

†Includes: *Streptococcus oralis* (4), *Streptococcus gordonii* (1), *Streptococcus mitis* (3), *Enterococcus faecium* (2)*, Streptococcus sanguinis* (1).

‡CNS, Includes: *Staphylococcus epidermidis* (19), *Staphylococcus hominis* (3), *Staphylococcus haemolyticus* (2), *Staphylococcus nepalensis* (1), *Staphylococcus warneri* (1).

§Includes: *Bacillus* sp (5), Diphtheroid sp (7), *Micrococcus* sp (8), *Rothia mucilaginosa* (1), *Paenibacillus glucanolyticus* (1), *Kocuria marina* (1).

¶Fungal isolates identified using culture and MALDI-TOF.

**HSV-1 and *Acanthamoeba* isolates identified using targeted PCR.

CIM, corneal impression membrane; CNS, coagulase-negative *Staphylococcus*; HSV-1, herpes simplex virus type 1; MALDI-TOF, matrix-assisted laser desorption ionization-time-of-flight mass spectrometry; MK, microbial keratitis.

**Table 4:** CIM samples with more than one isolate

CIMs with five isolates	
1	*Serratia marcescens*, *Enterococcus faecium*, *Staphylococcus aureus*, *Corynebacterium striatum*, *Staphylococcus epidermidis*
CIMs with four isolates	
2	*Morganella morganii*, *Streptococcus gordonii*, *S. epidermidis*, *Bacillus cereus*
CIMs with three isolates	
3	*Corynebacterium pseudodiphtheriticum*, *S. epidermidis*, *Staphylococcus hominis*
4	*Klebsiella variicola*, *Enterobacter cloacae*, *Citrobacter freundii*
5	*Paenibacillus glucanolyticus*, *Kocuria marina*, *S. epidermidis*
6	*Micrococcus luteus*, *Bacillus* sp, HSV-1
7	*Streptococcus pneumoniae*, *S. epidermidis*, HSV-1
CIMs with two isolates	
8	*Staphylococcus aureus*, *Streptococcus oralis*
9	*Morganella morganii*, *S. oralis*
10	*Corynebacterium propinquum*, *S. hominis*
11	*Bacillus pumilus*, *Streptococcus mitis*
12	*Morganella morganii*, *Staphylococcus haemolyticus*
13	*Enterococcus faecium*, HSV-1
14	*S. epidermidis*, *Micrococcus luteus*
15	*S. aureus*, HSV-1
16	*Sphingomonas paucimobilis*, *S. hominis*
17	*Aeromonas caviae*, *S. epidermidis*
18	*Staphylococcus warneri*, *Micrococcus luteus*

CIM, corneal impression membrane; HSV-1, herpes simplex virus type 1.

No association was found between ocular trauma, traditional medicine use, largest corneal ulcer diameter >5 mm or corneal perforation with positive isolates (χ^2^ p=0.49, 0.19, 0.32 and 0.69, respectively). All 12 patients who were HIV positive had a positive culture result (p=0.06). No significant difference was found between the isolation rate in those who had used an antimicrobial immediately prior to presentation (p=0.94).

### Minimum inhibitory concentrations

MIC data were available for all but seven of the *Streptococcus* isolates which could not be recovered from storage. The MICs for each of the main bacterial subgroups are demonstrated in online supplemental tables S1–S3. The fluoroquinolones had the lowest distribution of MICs of all the antimicrobials tested for all three groups of bacteria.

Methicillin resistance was present in none of the *S. aureus* isolates (n=7). All seven *S. aureus* isolates were deemed susceptible to all antimicrobials tested using topical and systemic breakpoints determined by EUCAST^29^ and had MICs below the reported first quartile corneal concentration for all antimicrobials tested (online supplemental table S1).

Online supplemental table S2 shows the MICs for *Streptococcus* (n=8). For the fluoroquinolones, using EUCAST breakpoints,^29^ susceptibilities of isolates were 37.5% to ciprofloxacin, 62.5% to ofloxacin and 62.5% to moxifloxacin. All isolates had MICs below the reported first quartile corneal concentration for moxifloxacin but not for ciprofloxacin (37.5%) and ofloxacin (50%).^24^ Using EUCAST breakpoints,^29^ susceptibilities to the glycopeptides (eg, vancomycin) and chloramphenicol were 75%–87.5% and 100%, respectively, although 37.5% of isolates had MICs within the reported achievable aqueous humour concentrations for chloramphenicol.^25^

Online supplemental table S3 summarises the MICs for Enterobacteriaceae spp (n=6). Fluoroquinolone susceptibility using EUCAST breakpoints^29^ was 66.7% to ciprofloxacin, 88.3% to ofloxacin and 50% to moxifloxacin. 83.5% of Enterobacteriaceae isolates had MICs below the reported first quartile corneal concentrations for each of the fluoroquinolones tested.^24^ 83.3% of isolates were susceptible to gentamicin using the EUCAST^29^ systemic breakpoint and all isolates had MICs below the reported first quartile corneal concentration for gentamicin.^26^ 83.3% of Enterobacteriaceae isolates were susceptible to chloramphenicol using the EUCAST^29^ topical breakpoint; however, all MICs were above or within the achievable reported aqueous humour concentration for chloramphenicol.

### PCR results

Eight (11.3%) participants were found to have HSV-1 detected from their CIM (table 3). Four of these participants had mixed bacterial and viral infections (table 4). Of the eight participants, seven patients had a corresponding positive HSV-1 conjunctival swab. The PCR cycle threshold (Ct) result for the participant who had a positive CIM and negative conjunctival swab for HSV-1 was borderline positive (35.6). No samples tested positive for *Acanthamoeba*. For the 12 patients who had a negative culture result, 9 patients had no evidence of bacterial DNA using 16S rRNA PCR. Mixed sequences were found for all three patients who had a negative culture but detectable bacterial DNA.

### WGS of Malawian *S. aureus* keratitis isolates

The baseline demographics, predisposing factors and examination findings of the seven patients that grew *S. aureus* are shown in online supplemental table S5.

#### Molecular classification of isolates

The core characteristics of the seven sequenced *S. aureus* isolates are presented in online supplemental table S6.

#### Virulence factors

Virulence genes previously associated with ocular *S. aureus* isolates or *S. aureus* keratitis^22 23^ were identified in the seven Malawian *S. aureus* keratitis isolates (online supplemental table S7). Genes encoding for adherence were found in all seven isolates with clumping factor A (*clfA*), fibronectin-binding protein A (*fnpA*) and serine-rich adhesin for platelets (*sraP*) present in all isolates. Number of evasion genes varied greatly between the isolates. The cysteine protease genes (*scpA*, *sspB* and *sspA*) were found in 100% of isolates. Staphylococcal enterotoxins were found in all but one of the isolates with varying profiles. Gamma and delta haemolysin genes were found in all seven isolates. The *lukF-*PV and *lukS-*PV gene encoding PVL were present in 3/7 (43%) and 2/7 (29%) of isolates, respectively. The methicillin resistance gene (*mecA*) was not identified in any of the seven isolates.

Of the 12 individual virulence genes proposed to be significantly enriched among *S. aureus* ocular isolates,^22^ *sed*, *sej*, *ser* were not found in any of the isolates, eight virulence genes (*Ψ-ent2*, *Ψ-ent1*, *seg*, *sei*, *sem*, *sen*, *seo*, *seu*) were consistently present in 5/7 (62.5%) of the isolates and one (*sev*) was present in 2/7 (29%) of the isolates. Of those virulence genes that were found to be enriched among non-ocular isolates,^22^ the cellular adhesion gene (*fnbB*) and cell wall surface anchor (*sraP*) were found to be present in all isolates, the superantigen *ssl8* gene in 3/7 (42.9%) isolates and the cell wall surface anchor protein (*sasG*) in 1/7 (14%) isolate (online supplemental table S6).

As the number of isolations of *S. aureus* was small, it was difficult to correlate the presence or absence of virulence factors with severity of disease due to the presence of multiple confounding host factors (presence of systemic immunosuppression, herpes zoster ophthalmicus (HZO), prior use of steroid eye drops, use of traditional medicine, etc). Two cases, case number 4 and 7, had comparatively lower numbers of evasion genes compared with the others (presence of 6 and 4 evasion genes, respectively, compared with 12–15 present in the remaining 5 cases), see online supplemental table S7. Of these, case 4 presented with a small ulcer size and good visual acuity whereas case 7 who had a background of HIV infection, HZO on presentation and who had received previous treatment with steroid eye drops presented with a much more severe MK picture. Case 5 had the highest number of virulence genes present and a severe clinical picture on presentation (visual acuity HM with a corneal ulcer measuring 6×5 mm with the presence of a hypopyon).

## Discussion

Clinical outcomes in MK depend on a complex interplay of host factors, microorganism virulence factors and the MIC of an antimicrobial agent against the respective microbe. Previous corneal sampling methods have relied on specialist equipment and skills. The CIM does not require specialist skills or equipment and has a higher presumed pathogen isolation rate compared with traditional scraping methods in a UK population.^13 14^ The CIM therefore, lends itself to being used in settings where such skills and equipment are unavailable.

In this study, we successfully demonstrated that the CIM could be used to obtain corneal samples and identify a possible causative microorganism using both culture and targeted PCR techniques in 83% of patients presenting with presumed MK in Malawi. We also demonstrated that it was feasible to collect such samples in a rural setting where immediate access to a microbiology laboratory was not possible.

The average time between symptom onset and presentation was longer than typically seen in the UK with ulcers being much larger on presentation (average major ulcer diameter on presentation in this study was 4 mm vs 2.14 mm seen in an average UK population).^13^ This isolation rate was higher than the 65.5% isolation rate that we demonstrated using the same CIM and sampling technique in a UK population and this is likely due to the longer presentation times, less antimicrobial use and more advanced disease on presentation.^14^

Due to a lack of specialist equipment, corneal samples are not routinely carried out in Malawi and therefore, the microbiological profile of MK is unknown. The CIM has previously been shown to increase isolations of presumed pathogenic microorganisms in MK compared with traditional scraping methods in a UK population^13 14^ and therefore direct comparisons between the CIM and traditional scraping methods in this population were not made. It could be argued that as the microbiological profile of MK in Malawi is unknown, the possibility that the CIM may not have detected all microorganisms that traditional corneal scrapes could have detected cannot be ruled out. That said, this study was carried out in the winter months of Malawi where the climate is typically cool and dry. Gram-positive bacteria were the most common microorganisms isolated in this population and this is consistent with other studies that have been carried out in temperate climates using traditional corneal scraping methods.^36^

Several predisposing factors are implicated in MK which differ between countries. In this study, only 15.5% of patients had a history of previous trauma which is lower than other studies carried out in other sub-Saharan African countries.^10 37 38^ This low incidence of trauma, together with the cool and dry season that this study was undertaken in may account for the absence of fungal infections. In studies in sub-Saharan Africa (Ghana, Tanzania), that used similar fungal culture methods (Sabouraud glucose agar plates) to this study, fungal keratitis accounts for most positive culture cases and it is therefore assumed more fungal infections would be identified in the hot and rainy season.^12 39^ The CIM has successfully identified fungal infections previously using the same sampling methodology and therefore this would not account for the fungal isolation rate in this study.^14^ In the future, the microbiological profile of MK in Malawi should be studied over a longer time period to better understand seasonal variability of pathogen prevalence throughout the year.

While contact lens wear is the most common risk factor in industrialised countries, trauma and ocular surface disease are more significant in low-resource settings.^40–43^ In this study, contact lens wear was non-existent and there were higher rates of trichiasis and traditional medicine use compared with other sub-Saharan MK studies.^10 44 45^ Traditional medicine use is widely practised in Malawi and can lead to damage to the ocular surface and delays in presentations to hospitals and clinics.^46^ The different microorganism spectrum seen in this population with low rates of *Pseudomonas* sp infection and high rates of mixed infections compared with those seen in the UK are likely to reflect these different predisposing factors^14^

The prevalence of HIV infection (17%) was higher than that reported for the general population in Malawi (10%).^47^ This observation is suggestive of increased susceptibility to MK among HIV-positive individuals and is consistent with that found by Burton *et al*,^10^ in Tanzania. Like Burton *et al*,^10^ we found no association between the type of MK and HIV although previous studies have indicated that HSV-1 and fungal keratitis are more prevalent in HIV-positive patients.^48 49^ This study does emphasise the argument made by Burton *et al* 10 for ophthalmologists offering MK patients referral to HIV testing services in such settings.

The percentage of CNS species and the numbers of samples obtained with more than one isolate in this study are similar to that seen using the CIM in a UK population and lead to the question of which of these are likely to be contributing to disease and which may either be contaminants or part of normal ocular flora in this population.^14^ The comparatively large surface area of the 12 mm diameter CIM used in this study compared with the median corneal ulcer size of 4×2.9 mm may favour the uptake of commensals from the patients’ unaffected cornea and use of a smaller diameter CIM may reduce the culture of these bacteria. The CIM is minimally invasive and therefore can be used to obtain corneal samples from healthy eyes. Further work, taking samples from asymptomatic or unaffected eyes of patients with MK may differentiate grades of pathogenic and non-genic microorganisms and inform future treatment strategies.

In this population, we demonstrated that the detection rate for HSV-1 was higher using the CIM than the conjunctival swab and this is in keeping with our previous work that demonstrated that the CIM is more likely to detect the presence of HSV-1 in suspected herpes simplex keratitis.^15^

The MIC is used to determine susceptibility criteria so that an appropriate antimicrobial treatment can be chosen. Ideally, the antimicrobial with the lowest MIC and an expectation to achieve a concentration above the MIC in the cornea should be selected. If the MIC of the used antimicrobial is below the first quartile concentration of the antimicrobial in the cornea, then it is likely that the microorganism is susceptible.^24^

The availability of antimicrobial eye drops in public health facilities in Malawi is erratic due to funding constraints. However, ciprofloxacin and gentamicin are the most available and these are the most prescribed antimicrobial agents for the treatment of MK. In this study, resistance was present to both agents in all bacterial groups other than *S. aureus*. Moxifloxacin offered the best coverage for both Gram-positive and Gram-negative isolates when susceptibility was determined using known antimicrobial first quartile concentrations whereas chloramphenicol offered the best coverage for all bacterial isolates when susceptibility was determined by EUCAST breakpoints. Although the numbers for each bacterial group are small and the results are limited to the dry season, these susceptibility data are the first of its kind to be reported on African keratitis isolates. Future research is needed to study antimicrobial resistance to topically available antimicrobials throughout the year in Malawi. It is also important to acknowledge the limitations of using in vitro sensitivity results based on systemic administration and more research is needed to study the bioavailability of antimicrobials reaching the cornea and aqueous and to establish ophthalmic breakpoints for topically applied antimicrobials.

In this study, we present the whole-genome sequences of seven Malawian *S. aureus* keratitis isolates. We observed a variety of virulence genes that have previously been associated with ocular *S. aureus* isolates or *S. aureus* keratitis.^22 23^ There are currently no studies looking at ocular *S. aureus* virulence factors in Africa to compare our data to and data concerning the virulence and pathogenesis of sub-Saharan Africa *S. aureus* clinical strains overall is limited. As the number of *S. aureus* keratitis isolates obtained in this study is small, and patients had multiple confounding host factors present, it is difficult to correlate the presence or absence of virulence factors with disease severity. Clinical outcome data were not collected as part of this study and therefore it is not possible to correlate the presence of virulence factors with clinical outcomes. Panton-Valentine leucocidin (PVL) gene expression was comparatively higher in the Malawian *S. aureus* keratitis isolates (3/7, 42.9%) than that seen in *S. aureus* keratitis isolates from the UK (9.5%).^50^ PVL is associated with poorer clinical outcomes in MK^50^ and is known to be high among clinical *S. aureus* strains in Africa (74% of methicillin-susceptible *S. aureus* isolates) compared with Europe (0.2%) and the USA (11.5%).^51–54^ Further investigation into the role of virulence factors in MK pathogenesis in Malawi is warranted.

In this study, we have demonstrated that the CIM sampling technique can feasibly be used to collect corneal samples from patients with suspected MK in a setting such as Malawi where access to specialist skills and equipment can be limited. Furthermore, we have demonstrated that corneal samples could feasibly be collected in rural settings where immediate access to a microbiology laboratory is not possible. Although this study is small in numbers and findings are limited to the dry season, it is the first study of its kind to characterise the microbiological profile of MK in Malawi. Results from this preliminary study support the use of the CIM in a larger study aiming to characterise the microbiological profile of MK in Malawi in a larger population over a longer time period and could also be feasibly used in other such settings to do the same. A better understanding of the causative microorganisms, microorganism susceptibilities to antimicrobials and microorganism virulence factors is greatly needed to improve patient outcomes and reduce corneal blindness in Malawi.

10.1136/bmjophth-2024-001682.supp2 Supplementary data

## Data Availability

Data are available upon reasonable request. The data that support the findings of this study are available from the corresponding author, TS, upon reasonable request.
