# Supplementary material for: Microbial keratitis in Southern Malawi: a microbiological pilot study
Source: BMJ Open Ophthalmol. 2024 Apr 22;9(1):e001682. doi: 10.1136/bmjophth-2024-001682 (PMC11043707; doi:10.1136/bmjophth-2024-001682)
Supplement: Supplementary data [file bmjophth-2024-001682supp001.pdf]

Supplementary data

Table S1: MICs to S. *aureus*																		
**Strain**	**Ciprofloxacin/ Ofloxacin**			**Moxifloxacin**			**Cefuroxime**			**Gentamicin**			**Vancomycin/ Teicoplanin**			**Chloramphenicol**		
	**MIC**	**S¹**	**S²**	**MIC**	**S¹**	**S²**	**MIC**	**S¹**	**S²**	**MIC**	**S¹**	**S²**	**MIC**	**S¹**	**S²**	**MIC**	**S¹**	**S²**
**1**	0.25 / 0.19	S	S	0.023	S	S	1.5	na	S	0.125	S	S	1.0 / 0.38	S	S	3.0	S	S
**2**	0.19 / 0.38	S	S	0.047	S	S	2.0	na	S	0.5	S	S	1.0 / 0.25	S	S	4.0	B	S
**3**	0.125/ 0.19	S	S	0.032	S	S	1.5	na	S	0.25	S	S	1.5 / 0.25	S	S	3.0	S	S
**4**	0.25 / 0.38	S	S	0.047	S	S	0.25	na	S	0.064	S	S	1.5 / 0.25	S	S	2.0	S	S
**5**	0.19 / 0.38	S	S	0.032	S	S	2.0	na	S	0.38	S	S	1.5 / 0.75	S	S	4.0	B	S
**6**	0.19 / 0.38	S	S	0.032	S	S	1.5	na	S	0.5	S	S	1.5 / 0.5	S	S	4.0	B	S
**7**	0.38 / 0.38	S	S	0.023	S	S	1.5	na	S	0.25	S	S	1.0 / 0.25	S	S	4.0	B	S
**Total susceptible (%):**		**100**	**100**		**100**	**100**		**na**	**100**		**100**	**100**		**100**	**100**			**100**

Data are in milligrams per litre (mg/L). S = Susceptible; R = Resistant; B = Borderline (within stated antimicrobial corneal concentration range)

S¹: Susceptibility determined using known antimicrobial 1st quartile corneal concentrations. If the MIC is below the reported 1st quarter corneal concentration for that antimicrobial, then it is reasonable to expect the bacterial to be inhibited by that antimicrobial and the strain is reported as susceptible. Although the reported corneal concentrations of antimicrobials are high, the associated standard deviations are high, meaning that the concentrations are not consistent around the mean. Because the actual data is not available in the listed references, the first quartile concentration has been extrapolated from the reported mean and standard deviation unless the median was available. Data is not available in articles to check normality. The pharmacokinetics of antimicrobials in corneal ulceration is unknown, so caution is needed in extrapolating data. Reported 1st quarter corneal concentrations for the antimicrobials are as follows: ciprofloxacin: 2.40-3.71mg/kg (24), ofloxacin: 4.73-6.79mg/kg (24), moxifloxacin: 5.11-9.62mg/kg (24), cefuroxime: no available information in the literature, gentamicin: 12.71mg/kg (only reported in rabbit eyes (33)), vancomycin: 33.1mg/kg (53), teicoplanin: 7.6mg/L (n=20, 0mg/L in 9 patients) (54); chloramphenicol: 3.5-6.7mg/kg (concentrations only available for aqueous humor and no 1st quartile concentration available (32)).

S²: Susceptibility determined by the breakpoint defined by EUCAST (25). Topical breakpoints are available for ciprofloxacin (1mg/L), ofloxacin (1mg/L) and chloramphenicol (16mg/L). For the other antimicrobials, systemic breakpoints were used: moxifloxacin (0.25mg/L), cefuroxime susceptibility was inferred from cefoxitin disc testing, gentamicin (2mg/L), teicoplanin (2mg/L) and vancomycin (2mg/L).

**Table S2: MICs to *Streptococcus sp.***																		
**Strain**	**Ciprofloxacin/ Ofloxacin**			**Moxifloxacin**			**Cefuroxime**			**Gentamicin**			**Vancomycin/ Teicoplanin**			**Chloramphenicol**		
	**MIC**	**S¹**	**S²**	**MIC**	**S¹**	**S²**	**MIC**	**S¹**	**S²**	**MIC**	**S¹**	**S²**	**MIC**	**S¹**	**S²**	**MIC**	**S¹**	**S²**
**1**	4.0 / 1.5	R / S	R / S	0.19	S	S	0.094	na	na	3.0	S	na	1.0 / 0.38	S	S	1.5	S	S
**2**	0.38 / 1.0	S	S	0.125	S	S	0.19	na	na	4.0	S	na	1.0 / 0.094	S	S	2.0	S	S
**3**	3.0 / 6.0	B / R	R	4.0	S	R	0.75	na	na	48	R	na	2.0 / 0.38	S	S	6.0	B	S
**4**	4.0 / 0.25	R / S	R / S	1.5	S	R	6.0	na	na	6.0	S	na	6.0 / 0.125	S	R / S	6.0	B	S
**5**	4.0 / 6.0	R	R	0.38	S	S	0.25	na	na	24	R	na	1.0 / 0.125	S	S	2.0	S	S
**6***	0.25 / 0.25	S	S	0.38	S	S	3.0	na	na	1.0	S	na	256 / 256	R	R	4.0	B	S
**7**	6.0 / 12.0	R	R	0.5	S	S	1.0	na	na	12	B	na	0.75 / 0.047	S	S	2.0	S	S
**8**	1.5 / 8.0	S / R	S / R	0.75	S	R	1.0	na	na	1.5	S	na	0.75 / 0.75	S	S	1.0	S	S
**Total susceptible (%)**		**37.5 / 50**	**37.5 / 62.5**		**100**	**62.5**		**na**	**na**		**62.5**	**na**		**87.5**	**75.0 / 87.5**		**62.5**	**100**

Data are in milligrams per litre (mg/L). S = Susceptible; R = Resistant; B = Borderline (within stated antimicrobial corneal concentration range)
S¹: Susceptibility determined using known antimicrobial 1st quartile corneal concentrations. If the MIC is below the reported 1st quarter corneal concentration for that antimicrobial, then it is reasonable to expect the bacterial to be inhibited by that antimicrobial and the strain is reported as susceptible. Although the reported corneal concentrations of antimicrobials are high, the associated standard deviations are high, meaning that the concentrations are not consistent around the mean. Because the actual data is not available in the listed references, the first quartile concentration has been extrapolated from the reported mean and standard deviation unless the median was available. Data is not available in articles to check normality. The pharmacokinetics of antimicrobials in corneal ulceration is unknown, so caution is needed in extrapolating data. Reported 1st quarter corneal concentrations for the antimicrobials are as follows: ciprofloxacin: 2.40-3.71mg/kg (24), ofloxacin: 4.73-6.79mg/kg (24), moxifloxacin: 5.11-9.62mg/kg (24), cefuroxime: no available information in the literature, gentamicin: 12.71mg/kg (only reported in rabbit eyes (33)), vancomycin: 33.1mg/kg (53), teicoplanin: 7.6mg/L (n=20, 0mg/L in 9 patients) (54); chloramphenicol: 3.5-6.7mg/kg (concentrations only available for aqueous humor and no 1st quartile concentration available (32)).
S²: Susceptibility determined by the breakpoint defined by EUCAST (25). Topical breakpoints are available for ciprofloxacin (1mg/L), ofloxacin (1mg/L) and chloramphenicol (16mg/L). For the other antimicrobials, systemic breakpoints were used: moxifloxacin (0.25mg/L), cefuroxime susceptibility was inferred from cefoxitin disc testing, gentamicin (2mg/L), teicoplanin (2mg/L) and vancomycin (2mg/L).

Table S3: MICs to *Enterobacteralaes sp.*																		
**Strain**	**Ciprofloxacin/ Ofloxacin**			**Moxifloxacin**			**Cefuroxime**			**Gentamicin**			**Vancomycin/ Teicoplanin**			**Chloramphenicol**		
	**MIC**	**S¹**	**S²**	**MIC**	**S¹**	**S²**	**MIC**	**S¹**	**S²**	**MIC**	**S¹**	**S²**	**MIC**	**S¹**	**S²**	**MIC**	**S¹**	**S²**
**1**	32 / 32	R	R	32	R	R	128	na	R	6.0	S	R	256 / 256	R	na	256	R	R
**2**	0.008 / 0.064	S	S	0.064	S	S	96	na	R	0.75	S	S	256 / 256	R	na	4.0	B	S
**3**	0.004 / 0.064	S	S	0.094	S	S	12	na	R	0.38	S	S	256 / 256	R	na	8.0	R	S
**4**	0.19 / 0.25	S	R / S	0.75	S	R	96	na	R	2.0	S	S	256 / 256	R	na	6.0	B	S
**5**	0.012 / 0.125	S	S	0.125	S	S	6.0	na	S	1.0	S	S	256 / 256	R	na	4.0	B	S
**6**	0.016 / 0.25	S	S	0.38	S	R	3.0	na	S	0.38	S	S	256 / 256	R	na	4.0	B	S
**Total susceptible (%)**		**83.3**	**66.7 / 83.3**		**83.3**	**50.0**		**na**	**33.3**		**100**	**83.3**		**0**	**na**		**0**	**83.3**

Data are in milligrams per litre (mg/L). S = Susceptible; R = Resistant; B = Borderline (within stated antimicrobial corneal concentration range)

S¹: Susceptibility determined using known antimicrobial 1st quartile corneal concentrations. If the MIC is below the reported 1st quarter corneal concentration for that antimicrobial, then it is reasonable to expect the bacterial to be inhibited by that antimicrobial and the strain is reported as susceptible. Although the reported corneal concentrations of antimicrobials are high, the associated standard deviations are high, meaning that the concentrations are not consistent around the mean. Because the actual data is not available in the listed references, the first quartile concentration has been extrapolated from the reported mean and standard deviation unless the median was available. Data is not available in articles to check normality. The pharmacokinetics of antimicrobials in corneal ulceration is unknown, so caution is needed in extrapolating data. Reported 1st quarter corneal concentrations for the antimicrobials are as follows: ciprofloxacin: 2.40-3.71mg/kg (24), ofloxacin: 4.73-6.79mg/kg (24), moxifloxacin: 5.11-9.62mg/kg (24), cefuroxime: no available information in the literature, gentamicin: 12.71mg/kg (only reported in rabbit eyes (33)), vancomycin: 33.1mg/kg (53), teicoplanin: 7.6mg/L (n=20, 0mg/L in 9 patients) (54); chloramphenicol: 3.5-6.7mg/kg (concentrations only available for aqueous humor and no 1st quartile concentration available (32)).

S²: Susceptibility determined by the breakpoint defined by EUCAST (25). Topical breakpoints are available for ciprofloxacin (1mg/L), ofloxacin (1mg/L) and chloramphenicol (16mg/L). For the other antimicrobials, systemic breakpoints were used: moxifloxacin (0.25mg/L), cefuroxime susceptibility was inferred from cefoxitin disc testing, gentamicin (2mg/L), teicoplanin (2mg/L) and vancomycin (2mg/L).

Table S4: Class, gene identity, function, reference genome and GeneID of the *S. aureus* virulence factor database used in this study

Functional class	Gene	Description	*S. aureus* reference genome	GeneID	Length
	mecA	Confers resistance to methicillin	7292 reference *Staphylococcus* sequences	WP_001801873.1	669 AA
**Adherence**	clfA*	Clumping factor A, fibrinogen binding protein	MW2	Q8NXJ1	946 AA
	fnpA*[$]	Fibronectin-binding protein A	MW2	Q8NUU7	1015 AA
	eap/map*	Truncated map-W protein	Mu50	BAB58100.1	476 AA
	sasG[$]	Cell wall surface anchor family protein	COL	A0A0H2WXE4	1243 AA
	sraP[$]	LPXTG Cell wall surface anchor family protein	COL	Q5HCP3	2261 AA
**Evasion**	scpA*	Cysteine protease Staphopain A	Mu50	P65825	388 AA
	sspB*	Cysteine proteinase Staphopain B	MW2	Q8NX99	393 AA
	sspA*	Glutamyl endopepidase V8	MW2	Q8NX98.1	327 AA
	coa*	Staphylocoagulase precursor	N315	BAB41444.1	658 AA
	seb*	Staphylococcal enterotoxin B	S6	AAA88550.1	266 AA
	hla/hly*	Alpha-hemolysin precursor	MW2	BAB94909.1	319 AA
	ψent1[#]	Enterotoxin *ψent*1	N315	BAB42913.1	133AA
	Ψent2[#]	Enterotoxin *ψent2*	N315	BAB42912.1	136 AA
	selo#	*Staphylococcal* enterotoxin O	RF122	CAI81390.1	254 AA
	seln#	*Staphylococcal* enterotoxin N	RF122	CAI81386.1	251 AA
	selm#	*Staphylococcal* enterotoxin M	N315	WP_000821658.1	239 AA
	sei#	*Staphylococcal* enterotoxin I	RF122	CAI81388.1	242 AA

	seg#	*Staphylococcal* enterotoxin G	RF122	CAI81385.1	205 AA
	selv#	*Staphylococcal* enterotoxin V	A900624	WP_043859283.1	239 AA
	seu#	*Staphylococcal* enterotoxin U	RF122	WP_000764692	261 AA
	sed#	*Staphylococcal* enterotoxin D precursor	RN4220	AAB06195.1	258 AA
	sej#	*Staphylococcal* enterotoxin J	2144 identical sequences	WP_000750881.1	268 AA
	ser#	*Staphylococcal* enterotoxin R	2018 identical sequences	WP_000932627.1	259 AA
	ssl8[$]	Superantigen-like protein 8	MW2	AUU67599.1	232 AA
**Invasion**	*hlgA**	Gamma-hemolysin chain II precursor	MW2	BAB96207.1	309 AA
	*hlgB**	Gamma-hemolysin component B	MW2	BAB96209.1	325 AA
	*hlgC**	Gamma-hemolysin component C	MW2	BAB96208.1	315 AA
	*hld**	Delta-hemolysin	MW2	BAB95824.1	44 AA
	*lukF-PV**	Panton-Valentine leukocidin precursor chain F	MW2	BAB95243.1	325 AA
	*lukS**	Panton-Valentine leukocidin precursor chain S	MW2	BAB95244.1	312 AA

**Virulence genes used by Afzal et al* (23).
[#]*Virulence genes associated with ocular S. aureus isolates* (22)
[$]*Virulence genes associated with non-ocular S. aureus isolates* (22).

Table S5: Baseline demographics, predisposing factors and examination findings of the seven patients with MK who had *S. aureus* isolated.

	**1**	**2**	**3**	**4**	**5**	**6**	**7**
**Baseline demographics and predisposing factors:**							
**Sex**	F	F	F	M	M	M	M
**HIV positive**	Yes	Unknown	No	Unknown	Yes	Unknown	Yes
**Previous history of MK**	Yes	No	No	No	No	No	No
**Topical eyedrop treatment prior to presentation**	No	Yes - tetracycline	No	No	No	No	Yes - dexamethasone
**Traditional medicine use**	no	Yes	No	No	No	No	Yes
**Examination findings:**							
**Snellen VA on presentation**	HM	6/60	6/60	6/6	HM	NM	HM
**Location of ulcer**	Central	Central	Peripheral	Peripheral	Central	Peripheral	Central
**Major diameter of ulcer (mm)**	1	NM	1	0.5	6	NM	11
**Minor diameter of ulcer (mm)**	1	NM	1	0.5	5	NM	11
**Ulcer depth**	1	1	2	1	NM	2	2
**Hypopyon**	No	No	No	No	Yes	No	No
**Other examination findings:**	-	-	-	-	HZO	-	HZO
**Mixed culture growth**	Yes*	Yes**	No	No	No	Yes***	No

NM: not measured.
HZO: Herpes Zoster Ophthalmicus
*Mixed growth with *Enterococcus faecium*, *Corynebacterium striatum*, *Serratia marcescens* and *S. epidermidis*
**Mixed growth with *S. oralis*.
***Mixed growth with HSV-1

Table S6: General genomic features of the seven Malawian *S. aureus* keratitis genomes in this study.

	**1**	**2**	**3**	**4**	**5**	**6**	**7**
**Total length (bp)**	3,198,350	2,884,721	2,796,874	2,832,020	2,850,395	2,925,823	2,808,597
**Contigs**	6	1	6	2	3	1	1
**Largest contig**	2,932,126	2,884,721	2,369,645	2,806,382	2,800,994	2,925,823	2,808,597
**N50 (bp)**	2,932,126	2,884,721	2,369,645	2,806,382	2,800,994	2,925,823	2,808,597
**GC (%)**	32.59	32.79	32.72	32.82	32.68	32.81	32.88
**Prophage Regions**	5	5	2	3	2	4	3
**Plasmids**	3	3	4	1	4	3	0
**MLST**	Unknown* 59 nearest STs	?ST-34*	Unknown Nearest ST-1940[#]	Unknown Nearest ST-4803[&]	?ST-1940[%]	Unknown[$] 8 nearest STs	?ST-152
***Spa* type**	t1130	t136	t3772	t064	t2554	t136	t355
***agr*-type**	III	III	I	I	I	III	I

*Imperfect hit with *aroE* (identity 99.8%, coverage 99.8%)

[#]Imperfect hit with *arcC* (identity 99.8%, coverage 99.8%) and *gmk* (identity 99.8%, coverage 99.8%)

[&]Imperfect hit with *gmk* (identity 99.5%, coverage 99.5%)

%Imperfect hit with *arcC* (identity 99.3%, coverage 99.3%) and *gmk* (identity 99.8%, coverage 99.8%)

[$]Imperfect hit with *arcC* (identity 99.6%, coverage 9.8%)

^Imperfect hit with *arcC* (identity 99.8%, coverage 99.8%), *aroE* (identity: 99.8%, coverage 99.8%) and *gmk* (identity 99.8%, coverage 99.8%)

Table S7: Presence or absence of known virulence genes associated with ocular *S. aureus* strains

Functional class	Gene	*S. aureus* genome							
		1	2	3	4	5	6	7	Total
	mecA	0	0	0	0	0	0	0	0
Adherence	*clfA**	1	1	1	1	1	1	1	7
	*fnpA**[$]	1	1	1	1	1	1	1	7
	*eap/map**	0	0	1	0	1	0	0	2
	sasG[$]	0	0	0	1	0	0	0	1
	sraP[$]	1	1	1	1	1	1	1	7
	Total	3	3	3	4	4	3	3	
Evasion	*scpA**	1	1	1	1	1	1	1	7
	*sspB**	1	1	1	1	1	1	1	7
	*sspA**	1	1	1	1	1	1	1	7
	*coa**	0	0	1	0	1	0	0	2
	*seb**	0	0	0	1	1	0	0	2
	*hla/hly**	1	1	1	1	1	1	1	7
	ψent1[#]	1	1	1	0	1	1	0	5
	Ψent2[#]	1	1	1	0	1	1	0	5
	selo#	1	1	0	0	0	1	0	3
	seln#	1	1	1	0	1	1	0	5
	selm#	1	1	1	0	1	1	0	5
	sei#	1	1	1	0	1	1	0	5
	seg#	1	1	1	0	1	1	0	5
	selv#	0	0	0	0	1	0	0	1
	seu#	1	1	1	0	1	1	0	5
	sed#	0	0	0	0	0	0	0	0
	sej#	0	0	0	0	0	0	0	0
	ser#	0	0	0	0	0	0	0	0
	ssl8[$]	0	0	1	1	1	0	0	3
	Total	12	12	13	6	15	12	4	
Invasion	*hlgA**	1	1	1	1	1	1	1	7
	*hlgB**	1	1	1	1	1	1	1	7
	*hlgC**	1	1	1	1	1	1	0	6
	*hld**	1	1	1	1	1	1	1	7

	lukF-PV*	1	0	1	0	0	0	1	3
	lukS*	1	0	0	0	0	0	1	2
	**Total**	6	4	5	4	4	4	5	
**Total number of virulence genes:**		**21**	**19**	**21**	**14**	**23**	**19**	**12**	

**Virulence genes used by Afzal et al* (23)

[#]*Virulence genes associated with ocular S. aureus isolates* (22)

[$]*Virulence genes associated with non-ocular S. aureus isolates* (22).
